# Characterization and spontaneous induction of urinary tract *Streptococcus anginosus* prophages

**DOI:** 10.1099/jgv.0.001407

**Published:** 2020-04-21

**Authors:** Bridget Brassil, Carine R. Mores, Alan J. Wolfe, Catherine Putonti

**Affiliations:** ^1^​ Department of Biology, Loyola University Chicago, Chicago, IL 60660, USA; ^2^​ Department of Microbiology and Immunology, Stritch School of Medicine, Loyola University Chicago, Maywood, IL 60153, USA; ^3^​ Department of Computer Science, Loyola University Chicago, Chicago, IL 60660, USA; ^4^​ Bioinformatics Program, Loyola University Chicago, Chicago, IL 60660, USA

**Keywords:** *Streptococcus anginosus*, bacteriophage, urinary tract

## Abstract

*
Streptococcus anginosus* is an often overlooked and understudied emerging pathogen inhabiting many areas of the human body. Through our sequencing of *S. anginosus* strains isolated from the female bladder microbiota, we detected numerous prophage sequences. Bioinformatic analysis of these sequences identified 17 distinct groups of *S. anginosus* prophages. The majority of these phages exhibit no sequence homology to previously characterized temperate or virulent phage sequences, indicating an unexplored diversity of *Streptococcus* phages. By culturing these bacterial isolates, we confirmed that the prophages of five of these groups are capable of induction. One of these putative phages was imaged, the first such evidence of an *S *. *anginosus* virus-like particle; it exhibits morphological characteristics of siphoviruses.

## Introduction

*Streptococcus anginosus* and other members of the *S. anginosus* group (* S. intermedius* and *S. constellatus *) are primarily commensal bacteria of the oral cavity, throat, gastrointestinal tract and vagina [1]. However, *S. anginosus* also has been found within the bladder [2], with strains from the vagina and urinary tract of the same individual belonging to essentially the same strain [3]. *S. anginosus* is now considered an emerging pathogen. It has been associated with infections of the respiratory tract [4–6], brain abscesses [7, 8], liver abscesses [9], and skin and soft tissue infections [10]. It also has been isolated from infections of the head and neck, central nervous system, gastrointestinal tract and blood [6, 9]. Furthermore, *S. anginosus* can cause infections of the genitourinary tract [6, 9, 11]. It has been associated with acute glomerulonephritis [12], bacterial vaginosis [13, 14], urge urinary incontinence [2] and urinary tract infections [15].

Previously, our group sequenced *S. anginosus* isolates from the urinary tract and vagina [3]. Our analysis of the genomes of these urinary isolates revealed that all harboured prophages. One strain, *S. anginosus* UMB0839, contained five prophage sequences [16]. At that time, no prophages had been described for the species. Subsequently, a bioinformatic study predicted prophage sequences within publicly available genomes of various *Streptococcus* species, including several *S. anginosus* strains [17]. Nevertheless, an in-depth analysis of *S. anginosus* prophages has yet to be conducted. Furthermore, the viability of these prophages remains unknown. We selected 14 *S *. *anginosus* strains from the urinary tract for complete genome sequencing and phage isolation, presenting here both novel prophage sequences and the first evidence of prophage induction for this species.

## *Streptococcus anginosus* Isolates of the Bladder

Catheterized urine samples were collected from women as part of prior IRB-approved studies [2, 18–20]. Bacteria were isolated from these samples using the expanded quantitative urine culture (EQUC) method [20] and stored at −80 °C. Fourteen strains identified as *S. anginosus* by MALDI-TOF MS were selected for whole genome sequencing. Freezer stocks for each of the strains were first streaked on Columbia CNA agar with 5 % sheep blood plates (BD 221353) and incubated at 35 °C in 5 % CO_2_ for 24 h. A single colony was then selected and grown in BHI liquid medium at 35 °C in 5 % CO_2_ for 24 h. DNA was extracted with the Qiagen DNeasy Blood and Tissue Kit, and DNA libraries were constructed (Nextera XT Library Prep Kit) and sequenced using the MiSeq Reagent Kit v2, producing 250 bp paired-end reads. The raw reads were trimmed using sickle v1.33 (https://github.com/najoshi/sickle) and assembled with SPAdes v3.11.1 [21] (parameters: ‘only-assembler’ option for k=55, 77, 99 and 127). Genome coverage was calculated using bbmap v34 (https://sourceforge.net/projects/bbmap/); the scripts bbwrap.sh and pileup.sh were used to map trimmed reads to the assemblies and compute average genome coverage. Genome annotations were performed using NCBI’s Prokaryotic Genome Annotation Pipeline v4.8 [22]. Genome sequences as well as raw reads have been deposited in GenBank’s Assembly and SRA databases, respectively. Table 1 lists the genome assembly statistics for each strain. Genome sizes ranged between 1.87 and 2.26 Mbp. This is within the range of sizes for publicly available genomes (52 genomes: 1.79–2.31 Mbp). The average GC content was 38.72 %. Again, this in on par with genomes of other strains of *S. anginosus *.

**Table 1.** Genome assembly statistics and the number of prophage sequences identified

Strain	Genome size (bp)	Number of contigs	N50	Accession number	Number of prophages
UMB0248	2,261,348	91	53 274	VYWV00000000	5
UMB0567	1,948,733	56	78 484	VYWU00000000	2
UMB0595	1,870,792	39	85 075	VYWR00000000	2
UMB0619	1,927,957	41	71 633	VYWQ00000000	2
UMB0622	1,969,304	49	118,764	VYWP00000000	3
UMB0633	1,943,307	44	116,838	VYWM00000000	2
UMB2128	2,204,898	105	43 663	VYVZ00000000	6
UMB3444	1,923,186	42	88 335	VYVX00000000	2
UMB4683	2,066,950	32	100,828	VYVW00000000	3
UMB4708	2,106,325	54	104,455	VYVU00000000	6
UMB7052	1,956,946	73	61 834	VYVS00000000	4
UMB8390	2,155,037	87	51 935	VYVP00000000	2
UMB8616	2,005,385	34	111,649	VYVM00000000	7
UMB8710	2,026,560	43	118,775	VYVJ00000000	1

## Predicted *S. anginosus* prophage sequences

The assembled genome sequences were investigated for prophage sequences using PHASTER [23] and VirSorter [24]. The two tools predicted the same prophage regions. VirSorter predictions were selected for further analysis. Fifty-five prophage sequences were identified by VirSorter amongst the 14 *S *. *anginosus* strains. Four of the prophage sequences identified were >100 kbp in length. Eight prophages were removed from further consideration as they were incomplete; they were predicted as ‘incomplete’ or ‘questionable’ by PHASTER and predicted with low confidence by VirSorter. The number of prophages per genome is listed in Table 1. Prophage sequences were clustered based upon sequence similarity to identify related phages between strains. This clustering was performed using USEARCH v11.0.667 [25] with the cluster_fast parameter and an identity threshold of 80 %, and the results were manually inspected. The 47 prophage sequences were clustered into 10 groups (*n*=40). The remaining seven prophages did not resemble any other prophage sequence from our *S. anginosus* genomes and thus were not included in a group. Each predicted prophage sequence was annotated using PATRIC [26], and groups were aligned using MAFFT v7.388 through Geneious Prime 2019.1.1 (Biomatters) with default parameters [27].

**Fig. 1.**
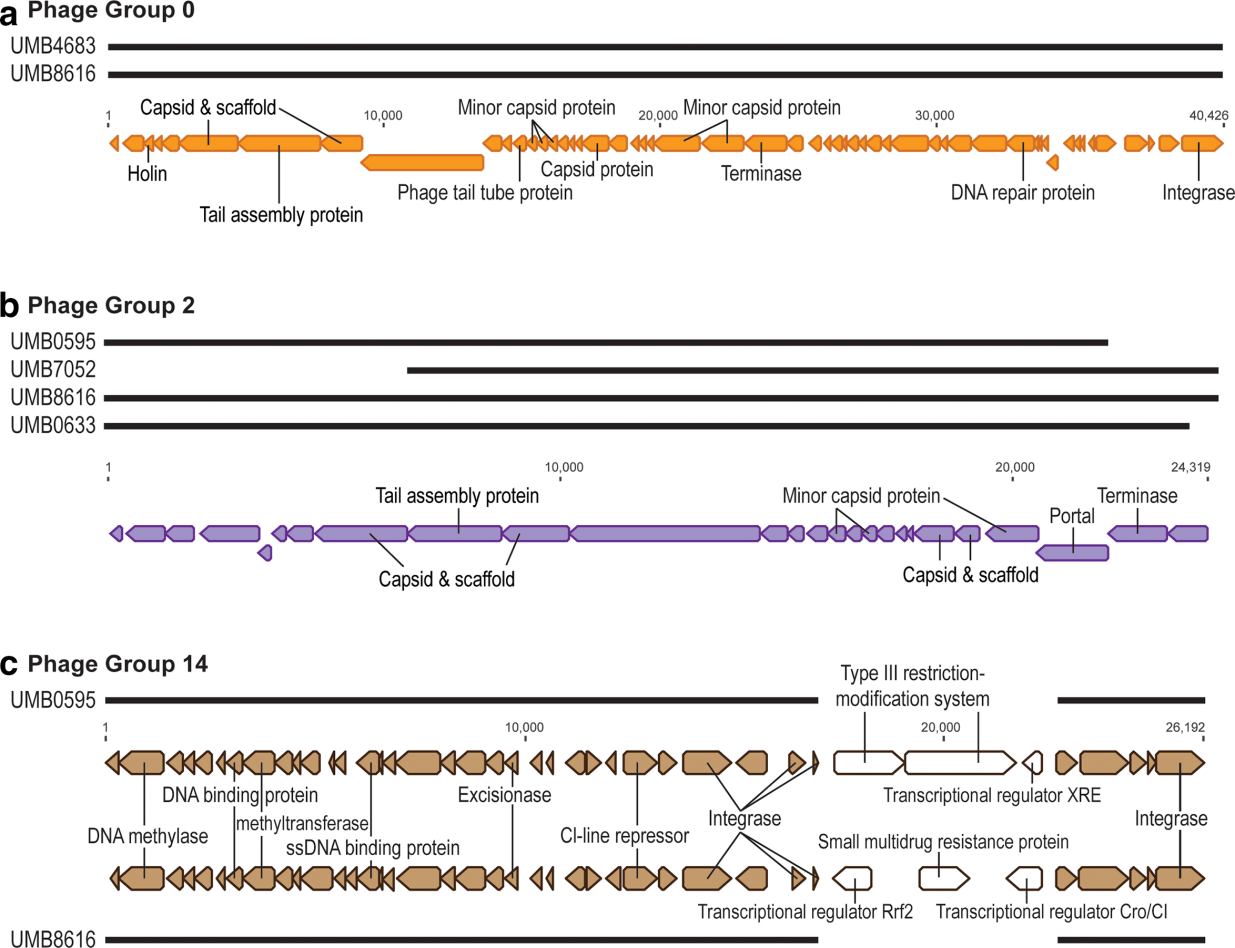
Gene annotations for predicted prophage sequences for phage groups 0 (a), 2 (b) and 14 (c). Predicted prophage sequence alignments are shown. Only two of the representatives of group 14 are shown. Unlabelled coding regions are annotated as ‘hypothetical proteins'.

Insertion sequences, integrases and tRNAs are frequently found to be flanking the predicted prophage sequences within the bacterial host genomes. Annotation details for all 47 prophage sequences can be found in Table S1 (available in the online version of this article). Inspection of these gene annotations reveals phage terminases, tail proteins, lysins and holins, capsid and scaffolding proteins, and integrases, as well as a multitude of hypothetical proteins. Fig. 1 shows the gene annotations for prophages from three of these groups. For group 14 (Fig. 1c), two of the four members are shown to highlight the variation that can exist within even related predicted prophages. While the majority of the prophage sequences are identical for this group, one region is unique: the prophage from UMB0595 codes for a Type III restriction-modification system and the prophage from UMB8616 codes for a small multidrug resistance protein. The sequences of group 7 prophages (141_10, 162_2 and 165_16), group 174 prophages (174_1-c4 and 174_1-c5) and the singleton prophage 161_8 code for tetracycline resistance. Other *Streptococcus *-infecting phages, including the *S. pyogenes* bacteriophage Φm46.1 (which has sequence similarity to the group 3 and group 7 phages) [28], have been found to carry tetracycline resistance genes. The group 7, group 174 and singleton 161_8 prophage sequences also encode a partitioning system. Similar systems have been identified in temperate phages of other hosts, enabling these phages to replicate extra-chromosomally [29] (and citations therein), [30]. Additionally, prophage 161_8 codes for the epsilon/zeta toxin–antitoxin system. Further experimental work is required to ascertain if the group 7, group 174 and singleton prophage 161_8 are in fact phages or rather phage-like mobile genetic elements (MGEs).

The 47 predicted prophage sequences were next compared to the NCBI nr/nt database of viruses and bacteria using megablast. All of the sequences most closely resembled annotated tailed *Streptococcus* prophages [17, 31], including prophages of *S. anginosus *, as well as *S. agalactiae *, *S. constellatus *, *S. crispatus*, *S. equi *, *S. gallolyticus *, *S. intermedius *, *S. mitis *, *S. oralis *, *S. pneumoniae *, *S. porcinus *, *S. thermophilus *, *S. uberis* and *S. urinalis *. Table 2 summarizes the blast similarities identified for the *S. anginosus* predicted prophage sequences. Full details of the blast analyses, including blast query results to the bacterial sequences of the nr/nt databases, can be found in Table S2. While most of the groups include prophage sequences with only modest sequence similarity to previously identified prophages, the prophages within groups 3 and 9 closely resemble the *S. agalactiae* prophage Javan 32 (GenBank: MK448906) and the *S. anginosus* prophage Javan 68 (GenBank: MK449004), respectively. Furthermore, the group 3 *S *. *anginosus* prophages and the *S. agalactiae* prophage Javan 32 also exhibit modest sequence similarity (43 % genome sequence coverage and 78.41 % sequence identity) to the characterized *S. pyogenes* phage Φm46.1 (GenBank: FM8642313). In a previous study, Φm46.1 was found to be capable of being transduced *in vitro* to strains of *S. agalactiae *, *S. gordonii* and *S. suis* [28]. This suggests that the group 3 phages may have a broad host range. With respect to the human urinary tract, this host range could include both the uropathogen *S. agalactiae* and *S. gordonii* [3, 32]. We have not found *S. pyogenes* or *S. suis* in urine samples of either men or women.

**Table 2.** Phage group demographics and similarity to previously characterized phages

Phage group number	Number of phages in group	Urinary host strains	Average prophage length (kbp)	Average query coverage (%)	Average sequence identity (%)	* Streptococcus* phage/prophage hit
0	2	UMB4683, UMB8616	37.4±1.8	31.00	86.85	Javan345 and Javan11
11	4	UMB0622, UMB2128, UMB7052, UMB7052	20.4±13.5	17.00	85.66	Abc2, Javan64, Javan648 and Javan355
14	4	UMB0595, UMB0633, UMB7052, UMB8616	19.4±9.3	19.75	94.83	IPP15
174	2	UMB8616, UMB8616	101.2±12.2	40.50	96.86	Javan618
2	4	UMB0595, UMB0633, UMB7052, UMB8616	21.6±2.9	24.25	92.23	Javan64 and Javan115
3	6	UMB0248, UMB2128, UMB4708, UMB4708, UMB8390, UMB8710	31.7±12.5	84.50	99.32	Javan32 and Javan618
4	8	UMB0248, UMB0619, UMB2128, UMB2128, UMB4683, UMB4708, UMB4708, UMB8616	31.0±23.5	24.50	92.87	Javan64, Javan68, Javan110 and Javan278
7	4	UMB0622, UMB2128, UMB4683, UMB4708	65.0±58.3	39.50	92.15	Javan426 and Javan638
8	3	UMB0248, UMB0567, UMB0619	60.7±44.5	3.00	86.31	Javan64 and Javan318
9	3	UMB0248, UMB0622, UMB8616	42.3±22.2	82.67	99.82	Javan68
Singleton 134_3	1	UMB0248	103.2	26.00	82.57	Javan112
Singleton 135_18	1	UMB0567	23.7	79.00	97.41	Javan64
Singleton 159_24	1	UMB2128	29.7	6.00	96.38	Javan104
Singleton 161_1	1	UMB3444	37.4	21.00	94.03	Javan226
Singleton 161_8	1	UMB3444	83.8	18.00	91.66	Javan638
Singleton 165_21	1	UMB4708	33.1	52.00	99.80	Javan38
Singleton 171_15	1	UMB8390	44.9	40.00	86.35	Javan191

## Evidence of Spontaneous Induction

Using the predicted prophage sequences, we designed primers to recognize prophages in groups 0, 2, 3, 4 and 14 (Table S3) to test for lytic phages induced spontaneously in liquid culture under laboratory conditions. The *S. anginosus* strains were first grown in BHI liquid medium at 35 °C in 5 % CO_2_. After 24 h of growth, 1 ml of the liquid culture was centrifuged at 13 000***g*** for 1 min, and the medium was filtered through a 0.2 µm cellulose acetate syringe filter. Lysates underwent a DNase treatment, using 1 U of OPTIZYME DNase I (Fisher BioReagents) following the manufacturer’s instructions. PCR was performed using 1 µl of the DNAsed lysates added to 1 µM of the forward and reverse primers, 25 µl PCR Master Mix (*Taq* DNA polymerase; Promega) and 22 µl nuclease-free water, for a final reaction volume of 50 µl. Amplification was conducted as follows: 95 °C denaturation temperature for 30 s (with an initial period of 5 min), 55 °C annealing temperature for 30 s, 72 °C elongation temperature for 30 s (with a final period of 10 min) and a total of 30 cycles. Additionally, the DNase-treated lysates underwent 16S rRNA gene PCR amplification, using primers 63f and 1387r [33] to check for bacterial DNA contamination. No 16S rRNA gene amplification was detected in any of the lysate PCRs. Following the above procedure, we conducted two trials of PCR-based detection (Table 3). Between these two trials, we detected the presence of all of the predicted prophages in groups 2, 4 and 14. Neither trial detected spontaneous induction for the group 3 phages in UMB8390 or UMB8710 or the group 0 phage in UMB4683. The PCR results for Trial 2 are shown in Fig. S1. While several of the prophages were detected in both trials, some were detected only in one of the trials, suggesting that spontaneous induction does not always occur or does not always occur at a detectable level. However, this is not surprising as the mechanisms triggering induction of some of the predicted prophages probably vary [34]. Nevertheless, detection of these predicted prophages in the medium suggests that prophages are capable of reproducing within the *S. anginosus* host.

**Table 3.** PCR detection of lytic phages for five of the identified groups +, Amplification; −, no amplification.

Phage group	Strain	Amplification	
		Trial 1	Trial 2
4	UMB0248	−	+
	UMB0619	+	+
	UMB2128	+	+
	UMB4683	−	+
	UMB4708	+	−
	UMB8616	+	+
2	UMB0595	+	+
	UMB0633	+	+
	UMB7052	+	+
	UMB8616	+	+
3	UMB0248	−	+
	UMB2128	−	+
	UMB4708	+	+
	UMB8390	−	−
	UMB8710	−	−
14	UMB0595	−	+
	UMB0633	−	+
	UMB7052	+	+
	UMB8616	+	−
0	UMB4683	−	−
	UMB8616	+	+

**Fig. 2.**
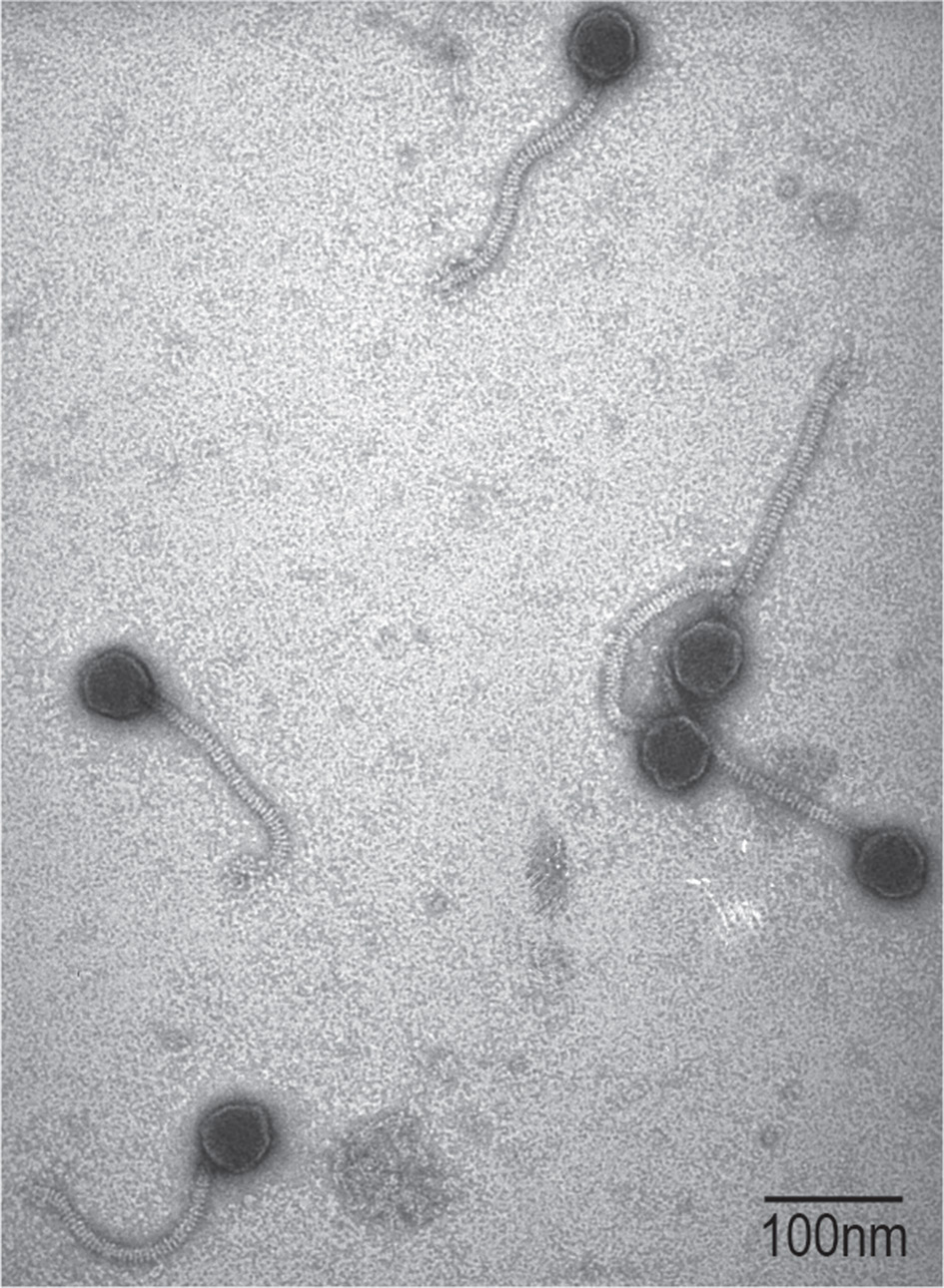
Transmission electron micrograph of virus-like particles induced from strain UMB4708. Bar, 100 nm.

In addition to PCR confirmation, lysates were prepared for microscopy. Lysate was applied to grids (Electron Microscopy Sciences) and negatively stained with 2 % uranyl acetate for visualization with a JEOL 1200 EX transmission electron microscope. Fig. 2 shows phages induced from the culture of *S. anginosus* UMB4708 from Trial 1 of the PCR screen. The observed morphology is representative of siphoviruses. While UMB4708 is predicted to contain six different prophage sequences (Table 1), the virus-like particles within the micrograph are quite homogeneous, having similar sized capsids and similar tail lengths. The transmission electron micrograph produced here shows similar morphology and size to the siphovirus Φm46.1 [35], which does exhibit sequence similarity to the group 3 prophage detected for UMB4708 (Table 3). As we also detected a group 4 prophage for this *S. anginosus* strain via PCR, we cannot definitively state which predicted prophage is imaged. Nevertheless, this is the first virus-like particle visualized for *S. anginosus *.

While PCR amplification and transmission electron micropscopy confirmed the presence of phage particles, we were unable to identify plaques for any PCR-positive phage lysates. Each PCR-positive phage lysate was plated on each urinary *S. anginosus* strain in our collection listed in Table 1. Plaquing was assessed using the soft overlay method where 300 µl of overnight bacterial culture and 100 µl of lysate were mixed with 1 ml BHI soft agar (0.5 % agarose). Spotting 10 µl lysate on bacterial lawns was also tested, including different host densities (300 µl to 1 ml). Plates were incubated at 35 °C in 5 % CO_2_. While we also spotted lysates from cultures grown with the inducing agents mitomycin C and H_2_O_2_, again plaques were not observed. The lack of observable plaques, however, does not necessarily mean that these phages cannot propagate through the lytic cycle. The availability of a naïve, domesticated laboratory strain will greatly benefit future investigation of *S. anginosus* phages in the lytic cycle. To the best of our knowledge, only one phage has previously been documented to infect an *S. anginosus* strain – the temperate phage P10 from an *S. oralis* strain [36]. This is, however, the first analysis of *S. anginosus* prophage sequences and the first evidence of putative *S. anginosus* phages. Phages capable of infecting this emerging pathogen have potential therapeutic applications given the documented antibiotic resistance of many strains [37]. Thus, further characterization and isolation of *S. anginosus* is greatly needed.

## Supplementary Data

Supplementary material 1

Supplementary material 2

Supplementary material 3
