# Supplementary material for: Characterization and spontaneous induction of urinary tract Streptococcus anginosus prophages
Source: J Gen Virol. 2020 Apr 21;101(6):685–91. doi: 10.1099/jgv.0.001407 (PMC7414447; doi:10.1099/jgv.0.001407)
Supplement: Supplementary material 3 [file jgv-101-685-s003.pdf]

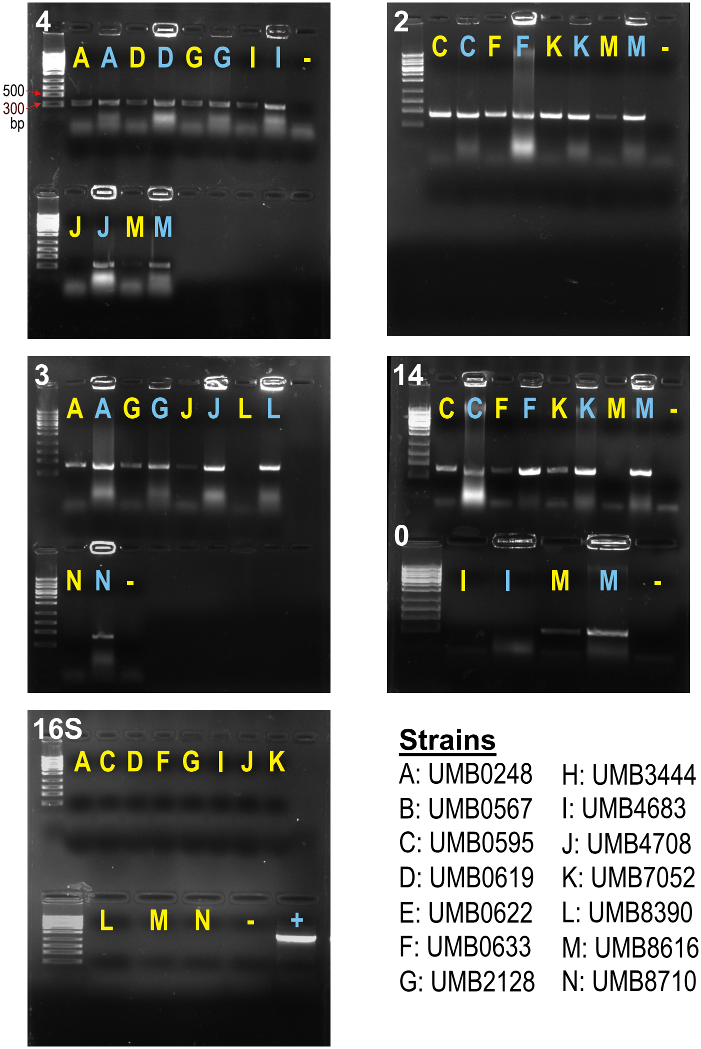

Supplemental Figure 1. Agarose gels for Trial 2 PCR-based detection of predicted prophage sequences in viral lysate (yellow) and bacterial colony controls (light blue). Negative controls (no DNA) are indicated by "-".

Supplemental Table 3. Primers for detecting lytic phages.

Phage Group #	Primer Pair	Expected Amplicon Size (bp)
2	CTGCAACCTCATCATTGC & CCGGCGCTGTCTTATATC	475
4	ATTCGCGCTAAGAAGTGC & TGCTCAGAATGTGCTTGG	349
3	ATTGCGCAAGGACAGC & CGAATTGGTGCGACTATG	460
14	TCGCTCAATCATCTCATCC & GATATGCCGGTCTTGGAG	447
0	TGAGCTTGCGTAGGTCAG & AGCGCAGACTCAGAGAGG	307
